# Development of a fluorescence-based assay for RecBCD activity using functional data analysis and design of experiments†

**DOI:** 10.1039/d4cb00291a

**Published:** 2025-03-17

**Authors:** Adam Winnifrith, Steven R. Brown, Piotr Jedryszek, C. Grant, Philip E. Kay, Adam M. Thomas, Jacob D. Bradbury, Thomas Lanyon-Hogg

**Affiliations:** a Department of Pharmacology, University of Oxford OX1 3QT UK thomas.lanyon-hogg@pharm.ox.ac.uk; b Synthace 4th Floor The Westworks, 195 Wood Lane London W12 7FQ UK; c Department of Biology, University of Oxford OX1 3SZ UK; d SAS Institute, Wittington House Henley Road Medmenham Marlow Buckinghamshire SL7 2EB UK; e Ineos Oxford Institute for Antimicrobial Research, University of Oxford OX1 3RE UK

## Abstract

Biochemical assays are essential tools in biological research and drug discovery, but optimisation of these assays is often a challenging and lengthy process due to the wide range of input variables and the complex effects of these variables on one another. Traditional ‘one-factor-at-a-time’ optimisation is both time-consuming and fails to explore the full range of input combinations. In contrast, the modern ‘design of experiments’ (DoE) approach enables simultaneous investigation of multiple input variables and their interactions, leading to more information-rich and efficient experimentation. We therefore sought to apply DoE to the optimisation of a new fluorescence-based assay for the enzyme RecBCD, a helicase–nuclease–ATPase complex involved in bacterial stress responses. A novel ‘functional data analysis’ (FDA) approach was used to predict the shape of RecBCD reaction curves in response to different combinations of input variables, which successfully identified assay conditions suitable for drug screening. Collectively, this work delivers a new assay for the antibiotic target RecBCD and demonstrates the potential of DoE and FDA to accelerate biochemical assay development.

## Abstract 2

This work describes the rapid optimisation of a new fluorescence-based assay for RecBCD enzyme activity by integrating D-optimal design of experiments, functional data analysis, and automation.

## Graphical abstract

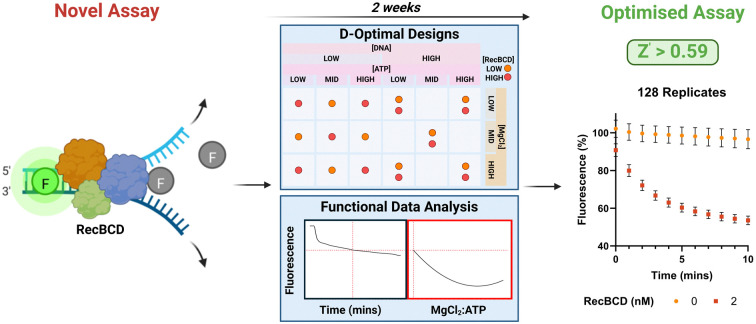

## Introduction

**Fig. 1:**
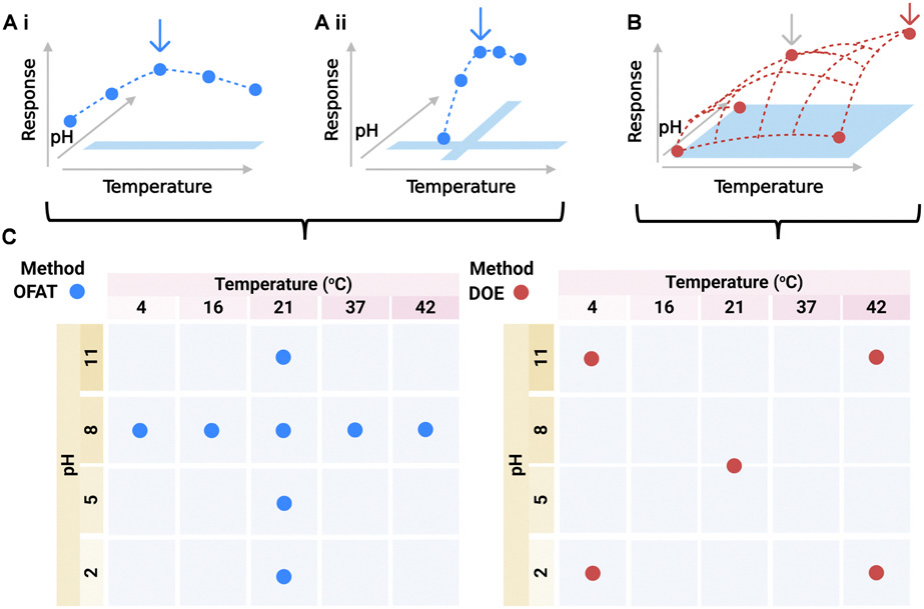
Assay optimisation strategies. (A) Schematic representation of a traditional ‘one-factor-at-a-time’ (OFAT) optimisation. In this example, (i) the first input factor (temperature) is varied with a fixed value of the second input factor (pH), and the optimal temperature found; (ii) the second input factor, pH, is varied at the selected temperature to identify the apparent optimal combination of conditions. (B) Design of experiments (DoE) explores the full range of design space for both factors simultaneously. In this example, DoE identifies the global optimum at both high pH and high temperature, which is overlooked by OFAT, and achieves this with fewer experiments and fewer conditions tested. (C) Exploration of input factor design space using OFAT and DoE strategies.

Analysis of complex biological systems is crucial for both fundamental research and drug discovery. Biochemical assays to study the function of individual proteins or protein complexes are essential tools in both contexts. However, the inherent complexity of these biochemical systems necessitates extensive effort in the design, analysis, and optimisation of assays due to the wide range of potential input variable combinations. The traditional approach to assay optimisation is by changing one variable (*e.g.*, temperature, pH, reagent concentration) and selecting the value that gives the optimal response, before changing the next variable (Fig. 1A). However, this ‘one-factor-at-a-time’ (OFAT) approach is both time-consuming and inefficient as only a limited proportion of the potential variable combinations is explored. Additionally, OFAT overlooks possible interactions between different experimental variables in the assay response.

‘Design of experiments’ (DoE) was developed in the mid-20th century for agriculture field trials and chemical engineering, and has since expanded into a versatile tool for process and product optimisation.^1,2^ DoE provides a mathematical framework to investigate the effect of multiple variables and their interactions by systematically changing all input variables simultaneously, and using statistical models to make predictions about how each individual variable affects the output response.^3,4^ By using statistical experimental design to determine the smallest set of conditions required to examine the effect of multiple factors with sufficient statistical power, DoE overcomes the challenge of needing to test all possible factor combinations.^5^ As a result, in contrast to OFAT exploration that follows one-dimensional lines in a multidimensional design space (Fig. 1A), DoE explores the overall response surface (Fig. 1B). DoE can enhance the information gathered from experiments and allow identification of optimal variable combinations potentially overlooked by OFAT, using fewer experimental conditions (Fig. 1C).

In DoE, independent experimental input variables whose values are controlled are termed ‘factors’, the values of factors selected for testing are termed ‘levels’, and the output dependent variables are termed ‘responses’. To suit different experimental needs and constraints, pre-calculated design strategies have been developed, which include but are not limited to: full factorial, fractional factorial, response surface, mixture, Taguchi array, and split plot designs.

DoE has been applied in industrial drug discovery^6^ to optimise cell based assays,^7,8^ enzyme linked immunosorbent assays,^9^ and recombinant protein production and purification,^4,10^ as well as in design of hybrid synthetic promoters in yeast.^11^ Yet despite successful implementation in industry, DoE lacks widespread adoption in academic research, where resource constraints mean efficient experimental design may yield most benefits.

The development of new medicines for bacterial infections is a field that would particularly benefit from more efficient academic research. Antimicrobial resistance (AMR) is one of the most serious threats to modern medicine and yet the current antibiotic pipeline is insufficient to meet this growing challenge due to both technical and economic challenges.^12^ Use of any new antibiotic is limited to mitigate resistance emergence; however, this disincentivises commercial antibiotic development, as low unit sales diminish the appeal for investors. Consequently, much early-stage antibiotic research is now conducted in academia, where resources and funding are limited.^13^ New methodologies that generate information-rich datasets rapidly, and with reduced resources, are therefore important to accelerate drug discovery efforts in this setting.

We therefore sought to use DoE to optimise a new biochemical assay for the emerging antibiotic target RecBCD, a DNA-damage repair enzyme that initiates the bacterial ‘SOS response’ and drives resistance evolution.^14^ D-optimal and space-filling DoE designs were used to rapidly identify factors affecting RecBCD activity and conditions that lead to a suitable assay signal for high-throughput screening.^2^ A novel ‘functional data analysis’ (FDA) approach was used to model the effect of different input factor combinations on the shape of the RecBCD assay response curve. The presented DoE workflow is also compatible with automation, digital experimental design, and machine learning, to conduct assay optimisation in days or weeks rather than months.

## Results and discussion

### Fluorescence-based assay for RecBCD activity

**Fig. 2:**
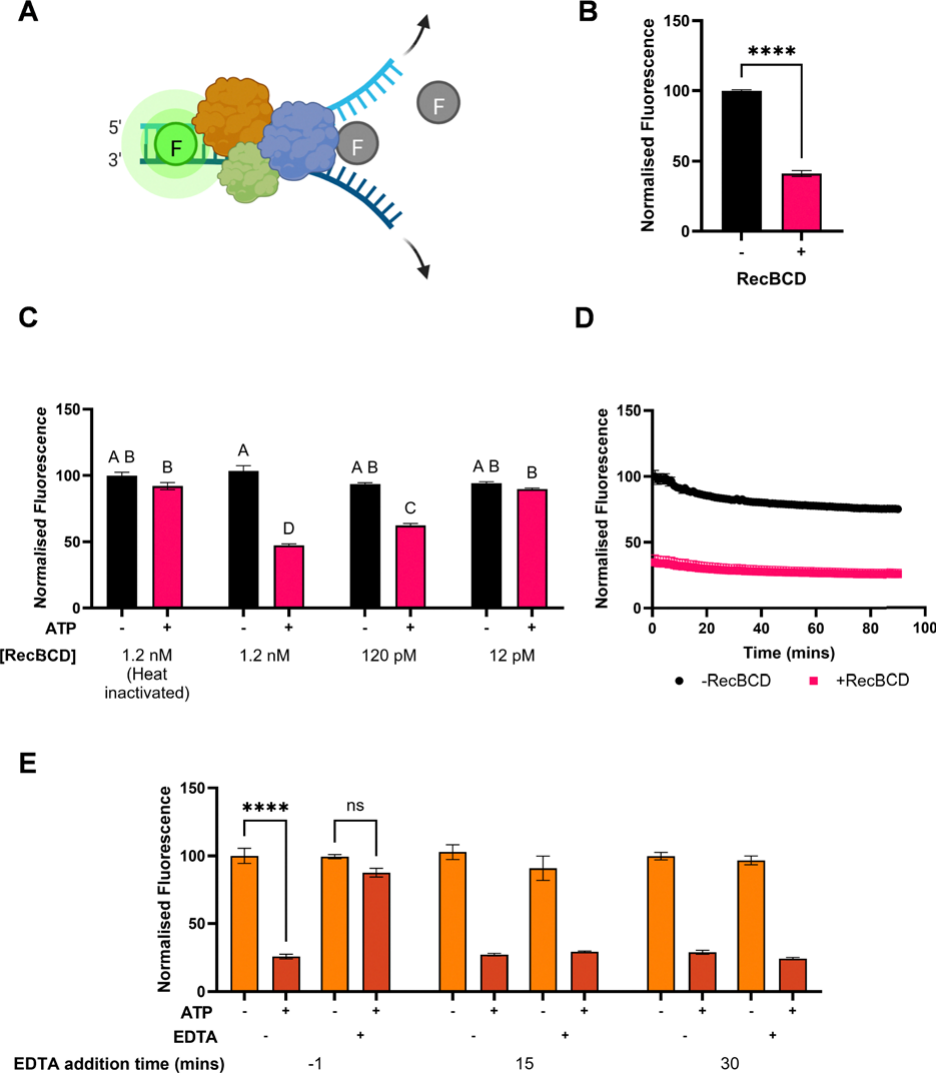
Fluorescence-based assay for RecBCD activity. (A) Schematic of the RecBCD assay. QuantiFluor dsDNA dye fluoresces when bound to dsDNA; RecBCD processing of dsDNA is detected as a decrease in fluorescence. (B) RecBCD activity measured as described in methods with RecBCD (4.8 nM), ATP (1 mM), Lambda DNA (5 ng μL^−1^), SSB (1.2 μM), and NEB Buffer 4 (1 ×). QuantiFluor (1 : 400 dilution, 50 μL) was added after 3 h. *t*-Test, ****P < 0.05. (C) RecBCD activity after 2 min was measured following heat inactivation at 95 °C for 30 min or in dose–response. The assay was performed as described in methods with varying concentrations of RecBCD with or without ATP (1 mM). Two-way ANOVA main effects (RecBCD and ATP) and interaction effect, multiple pairwise comparisons using Turkey's test; conditions sharing a common letter (A–D) were not statistically significantly different. (D) Assay signal measured kinetically over 90 min. The assay was performed as in B, but QuantiFluor (50 μL) was added immediately after initiating the reaction. (E) Inhibition of RecBCD activity by EDTA. Reactions were prepared as described in methods, with or without ATP (1 mM). EDTA was added to a final concentration of 0.1 M 1 min before, or 15 and 30 min after, addition of ATP. QuantiFluor (20 μL) was added after the final EDTA stop. Three-way ANOVA main effects (EDTA, ATP, time) and interaction effect, multiple pairwise comparisons using Turkey's test, ****P < 0.0001, ns, nonsignificant. Data represents means ± SEM, *n* ≥ 3.

RecBCD is helicase–nuclease enzyme complex that initiates repair of double-stranded DNA (dsDNA) breaks in bacteria and activates the mutagenic ‘SOS response’, making this enzyme an attractive therapeutic target.^14^ RecBCD separates dsDNA into single strands and cuts these DNA strands in an ATP-dependent manner.^15^ We therefore sought to establish a biochemical assay for RecBCD using the fluorogenic dsDNA dye QuantiFluor (Promega, US), where enzymatic processing of dsDNA would result in decreased fluorescence (Fig. 2A). A reaction mixture was prepared containing RecBCD (4.8 nM), ATP (1 mM), NEBuffer 4 (1×) and single stranded DNA binding protein (SSB, 1.2 μM) to prevent reannealing of DNA strands if reaction conditions promoted helicase activity over nuclease cutting. Following addition of QuantiFluor dye and Lambda DNA (5 ng μL^−1^) to initiate the reaction, a significant decrease in fluorescence was observed in the presence of RecBCD following 3 h incubation (Fig. 2B). The inability of RecBCD to fully remove all double-stranded Lambda DNA signal under these assay conditions may result from the complementary 12 base-pair overhangs on the ends of double-stranded Lambda DNA, which would result in a certain proportion of cyclic dsDNA that cannot be processed by RecBCD. To confirm assay signal was dependent on enzyme activity, RecBCD was heat-inactivated, resulting in abrogation of fluorescence decrease (Fig. 2C). RecBCD contains two ATP-dependent helicase subunits, and correspondingly no change in signal was observed in the absence of ATP; assay signal in the presence of ATP was also dependant on RecBCD concentration (Fig. 2C).

Real-time measurements of fluorescence for 90 min following initiation of the RecBCD reaction revealed little change in signal over time, suggesting the enzymatic activity had reached completion before the first measurement (Fig. 2D). Given the known rapid processivity of RecBCD,^16,17^ stop conditions were therefore sought to confirm the time dependence of the dsDNA fluorescent assay signal. EDTA inhibits RecBCD through chelation of Mg^2+^ ions essential for helicase^18,19^ and nuclease activity.^20^ Addition of EDTA (0.1 M) before the enzyme reaction was initiated resulted in no decrease in fluorescence signal compared to no-ATP controls; EDTA addition 15 or 30 min after initiation of the enzyme reaction resulted in decreased fluorescence only in the presence of ATP (Fig. 2E). Inhibition of RecBCD activity by EDTA was dose-dependent with IC_50_ = 7 mM (Fig. S1, ESI†). Alternative DNA substrates were also tested in the assay; Lambda DNA provided the best signal window compared to other tested DNA substrates (Fig. S2, ESI†) and was therefore used in subsequent experiments. The QuantiFluor dsDNA signal was also stable to dimethyl sulfoxide (DMSO) concentrations <10% (v/v) typically used in drug discovery assays (Fig. S3, ESI†).

### DoE exploration of kinetic data

Having demonstrated that the QuantiFluor dsDNA fluorescence-based assay can assess RecBCD activity, but that the enzyme activity was too rapid for inhibitor screening, we employed a constrained D-optimal design of experiments approach to analyse the effect of different input factors and optimise the assay response. A D-optimal design algorithm selects an optimal set of conditions by maximising the determinant of the information matrix, thereby minimising parameter estimate uncertainty. Designs were generated within input factor constraints using either JMP or Synthace software platforms, ensuring the design was both statistically efficient and feasible to execute.

**Fig. 3:**
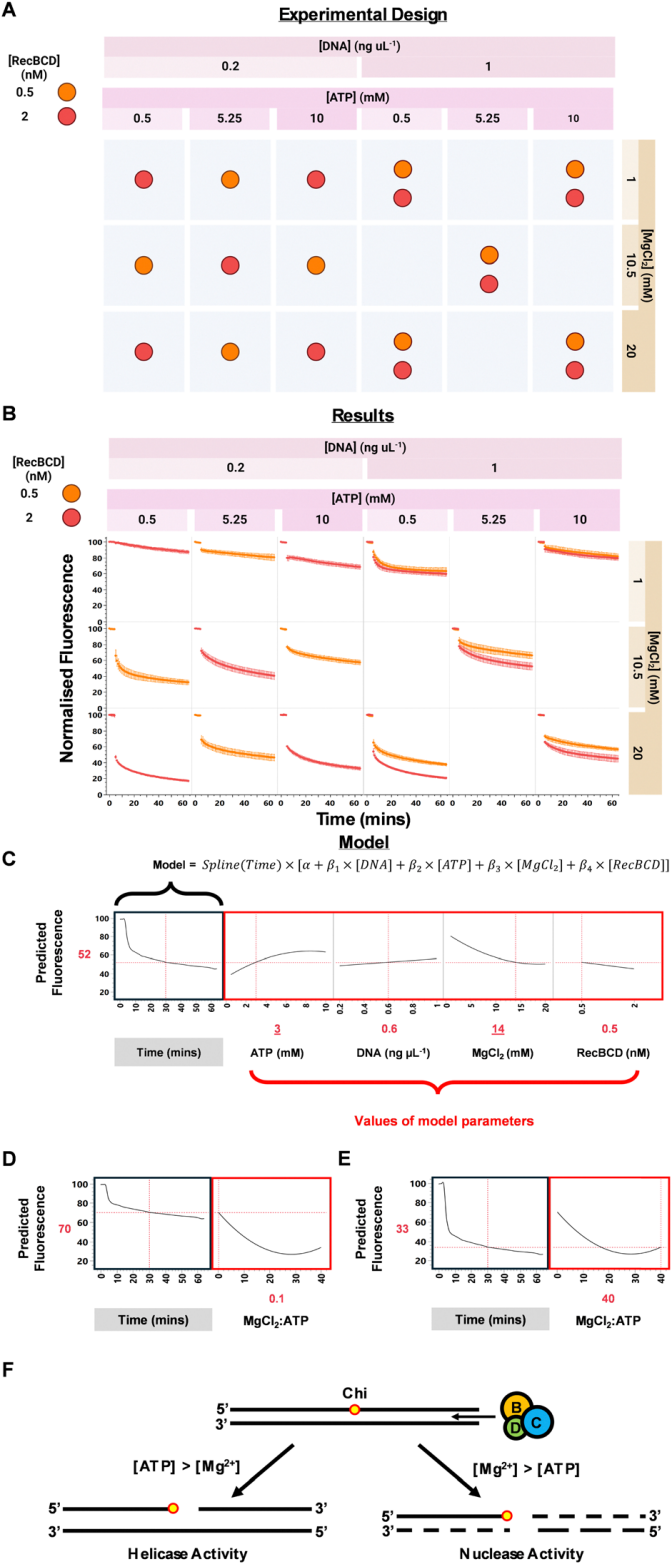
Design of experiments (DoE) and functional data analysis (FDA) exploration of RecBCD activity. (A) Experimental design matrix showing combinations of RecBCD, DNA, ATP, and MgCl_2_ concentrations tested. (B) Time-course fluorescence results for each condition. (C) FDA model predicting fluorescence based on input factors. (D) and (E) FDA model predictions showing the effect of MgCl_2_ : ATP ratio on fluorescence decrease: (D) low MgCl_2_ : ATP ratio (1 : 10), (E) high MgCl_2_ : ATP ratio (20 : 1). (F) Schematic illustrating how MgCl_2_ : ATP ratio affects RecBCD activity, favouring helicase or nuclease activity, adapted from ref. 15.

Initially, a four-parameter experimental design was generated with RecBCD (0.5–2 nM), DNA (0.2–1 ng μL^−1^), MgCl_2_ (1–20 mM), and ATP (0.5–10 mM) as input factors, resulting in 19 unique conditions covering the four-dimensional experimental design space (Fig. 3A and Table S1, ESI†). Each condition was tested with 3–6 replicates, with fluorescence recorded for 5 min prior to ATP addition and then for a subsequent 60 min after addition (Fig. 3B).

Functional data analysis (FDA)^21,22^ was used to model how the input factor combinations affected the shape of the assay response curve. Unlike kinetic analyses that reduce time-course data to single summary statistics (*e.g.* initial reaction rates or area under the curve), FDA models the entire enzyme reaction curve shape in response to different input factor levels. FDA therefore allows creation of interactive plots that predict the complete assay response curve for any combination of input factors (Fig. 3C). The JMP interactive Prediction Profiler was used to visualise the effect of different combinations of input factors on the response curve shape (Video S1, ESI†).

The FDA model generated insights consistent with previous validation experiments (Fig. 2), including that increased RecBCD generated a larger signal window between conditions with and without RecBCD (Fig. 3C). The model also showed that higher DNA concentrations increased raw fluorescence, while higher MgCl_2_ concentrations decreased fluorescence across the entire curve, possibly due to Mg^2+^ interference with dye fluorescence (Fig. S4, ESI†).

Further, FDA was capable of identifying two-factor interactions in the ratio of MgCl_2_ : ATP concentration that affected the response curve shape. Increasing the MgCl_2_ : ATP ratio led to a greater decrease in fluorescence, consistent with enhanced dsDNA degradation. When MgCl_2_ = 1 mM and ATP = 10 mM (ratio 0.1), the FDA model predicted a 30% decrease in normalised fluorescence after 30 min (Fig. 3D). In contrast, when MgCl_2_ = 20 mM and ATP = 1 mM (ratio 20), a 67% decrease in normalised fluorescence after 30 min was predicted (Fig. 3E). This MgCl_2_ : ATP ratio effect is consistent with previous literature on RecBCD function.^15,23–25^ When ATP concentration exceeds Mg^2+^, RecBCD primarily exhibits helicase activity; conversely, when Mg^2+^ concentration is higher, nuclease activity is favoured (Fig. 3F). This modulation is thought to result from ATP coordinating Mg^2+^ at low Mg^2+^ : ATP ratios, with Mg^2+^ being essential for nuclease activity.^15^

### High-dimensional experimentation DoE

**Fig. 4:**
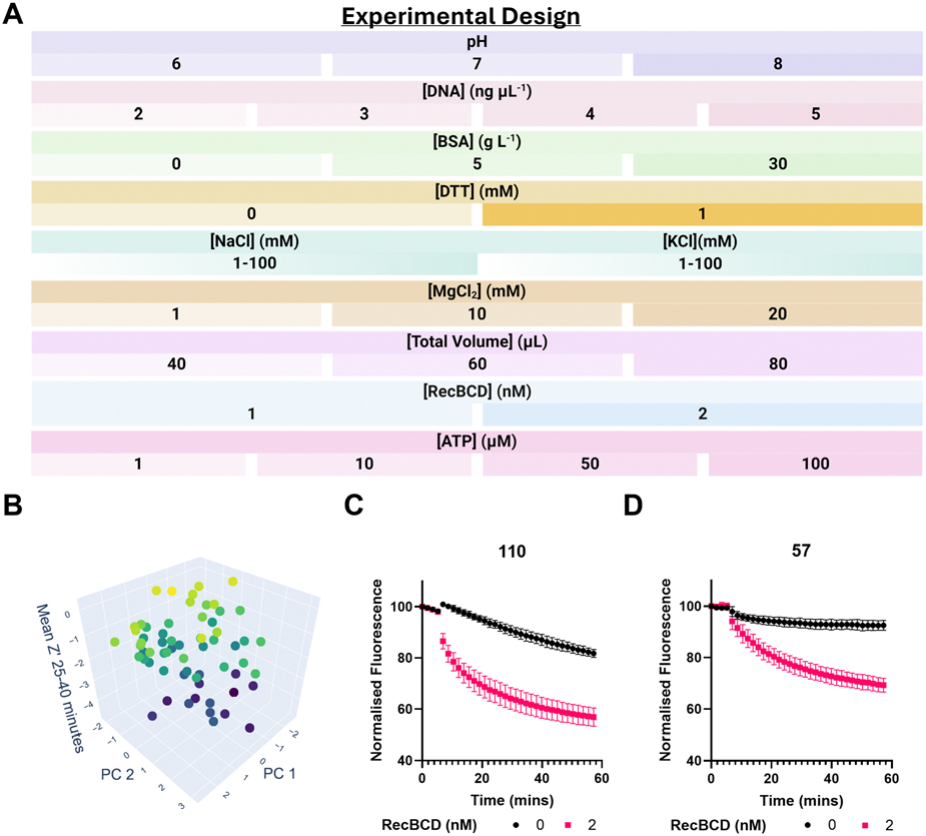
High-dimensional experimentation exploration of RecBCD assay. (A) Conditions explored within the experimental design space. Assay were performed as described in the methods. (B) Principal component analysis (PCA) of 10 factors from A plotted against mean *Z*′ (25–40 min) for each condition. Data points are coloured based on mean *Z*′ from blue (low *Z*′) to yellow (high *Z*′). Conditions with *Z*′ < −5 excluded. (C) and (D) Highest *Z*′ conditions. Data represents means ± SD, *n* ≥ 3.

To explore a broader range of variables on the assay signal, ten input factors which potentially impacted enzyme activity were investigated (Fig. 4A and Table S2, ESI†).^26^ The design criteria covered input factors RecBCD (0–2 nM), DNA (2–5 ng μL^−1^), MgCl_2_ (1–20 mM), ATP (1–100 μM), pH (6–8), BSA (0–30 g L^−1^), DTT (0–1 mM), NaCl *vs*. KCl, monovalent cation chloride salt concentration (1–100 mM), and reaction volume (40–80 μL, Fig. 4A).

120 combinations of input factors were tested in triplicate, with positive (+RecBCD) and negative (−RecBCD) controls. A space-filling design was used to evenly cover the whole experimental design space, ensuring that all possible combinations of factors were adequately represented (Fig. S5, ESI†). This method is particularly useful when exploring a high-dimensional space, as it maximises the diversity of conditions tested, helping to identify interactions and nonlinear effects. Experimental conditions were also randomised across the assay plate to minimise edge effects.

Most conditions showed no change in fluorescence (Fig. S6, ESI†); however, FDA identified trends such as >10 μM ATP being required for assay signal, in line with previous reports.^27^*Z*′ is a statistical measure of assay quality for high-throughput screening, with *Z*′ ≥ 0.5 indicating an excellent assay.^28^ To identify assay conditions compatible with small molecule screening, the *Z*′ was calculated for each condition and timepoint (see ESI†), with mean *Z*′ (25–40 min) used for ranking.

Principal component analysis (PCA) of the ten input factors against mean *Z*′ revealed that whilst most conditions had *Z*′ < 0, there were 8 conditions with *Z*′ > 0 spread across the design space (Fig. 4B). ‘Hit’ conditions 110 (*Z*′ = 0.32) and 57 (*Z*′ = 0.25) were selected for further optimisation (Fig. 4C and D, see Table S2 for details of conditions, ESI†). In condition 110, the decrease in no RecBCD fluorescent signal over time may result from quenching of dye fluorescence under the assay conditions.

### DoE analysis of local areas of design space

**Fig. 5:**
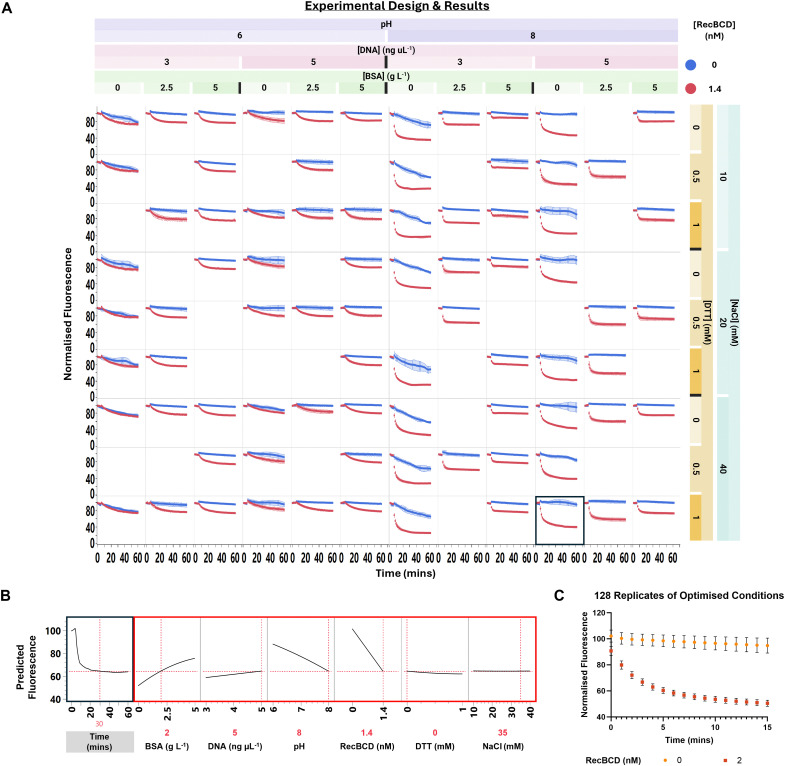
Optimisation and validation of RecBCD assay conditions. (A) Experimental design and results of localized DoE experiment. Normalised fluorescence plotted against time for 95 variations of conditions 110 and 57, focusing on DNA (3, 5 ng μL^−1^), pH (6, 8), BSA (0, 2.5, 5 g L^−1^), DTT (0, 0.5, 1 mM), and NaCl (10, 20, 40 mM). Results show the effects of these variables on RecBCD assay signal, with best performing condition 82 boxed in bold. Data represents means ± SD, *n* ≥ 4. (B) Functional data analysis model of raw data from (A), showing effects of BSA, pH, and DNA on assay signal. (C) Validation of best performing condition 82 (DNA = 5 ng μL^−1^, BSA = 0 mg mL^−1^, pH = 8, NaCl = 40 mM, DTT = 1 mM) with 128 replicates of positive and negative controls. Data represents means ± SD.

Conditions 110 and 57 had different concentrations of DTT (1 *vs*. 0 mM), BSA (0 *vs*. 5 mg mL^−1^), NaCl (37 *vs*. 11 mM), DNA (5 *vs*. 3 ng μL^−1^), and pH (8 *vs*. 6), respectively. As the number of factors was reduced and their levels more well-defined, a constrained D-optimal design was used to systematically explore the local design space around these two conditions (Fig. S7, ESI†). 95 factor combinations were tested with input factors DNA (3, 5 ng μL^−1^), pH (6, 8), BSA (0, 2.5, 5 g L^−1^), DTT (0, 1 mM), and NaCl (10, 20, 40 mM), with four replicates each (Fig. 5A and Table S3, ESI†). The MgCl_2_ : ATP ratio was fixed at 200 : 1 (10 mM MgCl_2_, 50 μM ATP) to promote nuclease activity, as the nuclease activity of RecBCD requires both helicase and ATPase functions and can therefore be used as a readout when screening for inhibitors of any of these RecBCD functions.

The focused local design space yielded 72% of conditions with *Z*′ >0 (Fig. 5A), compared to 8% in the high-dimensional screen of conditions (Fig. S6, ESI†). FDA indicated BSA, pH, and DNA concentration had the largest effects on assay signal (Fig. 5B): pH 6 reduced RecBCD activity, BSA stabilised fluorescence at low DNA concentrations in the absence of RecBCD, and high BSA concentrations decreased the assay signal window between positive (+RecBCD) and negative (−RecBCD) controls (Video S2, ESI†). DTT and NaCl concentration had minimal effects on RecBCD activity. To check consistency between FDA and other statistical methods, a random forest model was constructed to predict fluorescence at 30 min based on RecBCD and the DoE factors. This model indicated RecBCD, BSA, and pH had feature importances of 53%, 23%, and 16%, respectively (*R*^2^ = 0.92, root mean squared error {RMSE} = 5.5, Fig. S8, ESI†). A linear model of the same prediction task showed significant interactions between pH and RecBCD (*p* = 0.0058), BSA and RecBCD (*p* = 0.0023), and pH and BSA-RecBCD (*p* = 0.00077), suggesting RecBCD activity was sensitive to pH and BSA levels.^20,29^ To check the robustness of the optimised assay condition, the best performing condition was repeated with 128 replicates, yielding a *Z*′ after 10 min of 0.59 (Fig. 5C), highlighting the reproducibility of the assay.

## Conclusions

Biochemical assays present challenges in their analysis and optimisation due to the scale and interactions of potential input factors. This complexity makes assay optimisation both time consuming and expensive. To help address this challenge, we sought to employ a combination of DoE, automation, and digital experiment design to explore complex design spaces in a biochemical assay. As a test-case, we developed a new fluorescence-based assay for the helicase–nuclease enzyme RecBCD, an emerging target in antibiotic discovery.^30–33^

A single four-parameter DoE was capable of identifying conditions that promoted RecBCD nuclease activity at increased Mg^2+^:ATP ratios (Fig. 3), consistent with prior literature using OFAT analysis.^15,20,23,29^ Under reaction conditions where RecBCD was active, the reaction plateaued with 40% of the starting fluorescence signal remaining. This may suggest the presence of remaining dsDNA, potentially as a result of circular Lambda DNA formed from complimentary base pairs. Boiling of Lambda DNA to produce fully linear dsDNA prior to initiation of the RecBCD reaction may therefore provide a larger signal window and also increase the enzyme reaction kinetics. Expanding DoE analysis to ten input factors allowed screening of multiple conditions simultaneously and identified factors with no impact on assay readout as well as assay conditions yielding *Z*′ > 0. A focused exploration of ‘hit’ conditions identified a reaction set up with robust assay performance (*Z*′ = 0.59 with 128 replicates).

We build on previous literature applying DoE to assay optimisation in two ways. Firstly, we introduce the application of functional data analysis (FDA) to modelling and optimising kinetic responses. FDA allows prediction of the effect of different input factor combinations on the response curve shape, offering insights into assay behaviour and enabling optimisation towards specific reaction profiles, such as linear reaction rates. Secondly, we combine D-optimal designs, automation, and digital experiment platforms to rapidly explore large areas of assay design space, identifying complex, multi-dimensional assay response surfaces with distinct optima. A key advantage of this approach was the ability to design and implement high-dimensional DoE experiments within one week using Synthace's digital experiment platforms, which integrate DoE designs, liquid handling instructions, experiment simulations, and metadata tracking. This platform is also compatible with incorporation of reagent costs, such that an assay can be optimised to maximise signal within the available financial budget for reagents.

The presented experimental approach therefore provides advantages in efficiency of both data gathering and data interpretation compared to conventional OFAT and DoE optimisation. Widespread adoption could substantially impact biochemical assay optimisation and drug discovery, particularly in resource-limited settings such as antibiotic development.

## Author contributions

A. W., S. B., C. G., and T. L.-H. designed experiments. A. W., P. J., and P. K. analysed data. A. W. and S. B. performed biochemical testing. T. L.-H. was responsible for funding acquisition. S. B., C. G., A. M. T., J. D. B. and T. L.-H. supervised the research. A. W., J. D. B. and T. L.-H. wrote the manuscript with input from all authors.

## Data availability

The data supporting this article have been included as part of the ESI.†

## Conflicts of interest

S. R. M. and C. G. are employees of Synthace LTD, UK. P. E. K. is an employee of JMP Statistical Discovery LLC, US. A. W. and P. J. are founders of and hold shares in Evolvere Biosciences LTD, UK.

## Supplementary Material

CB-006-D4CB00291A-s001

CB-006-D4CB00291A-s002

CB-006-D4CB00291A-s003

CB-006-D4CB00291A-s004

CB-006-D4CB00291A-s005
