# Supplementary material for: Development of a fluorescence-based assay for RecBCD activity using functional data analysis and design of experiments
Source: RSC Chem Biol. 2025 Mar 17;6(5):772–9. doi: 10.1039/d4cb00291a (PMC11955833; doi:10.1039/d4cb00291a)
Supplement: CB-006-D4CB00291A-s005 [file CB-006-D4CB00291A-s005.pdf]

Supplementary Information (SI) for RSC Chemical Biology.
This journal is © The Royal Society of Chemistry 2025

Supporting Information

Development of a fluorescence-based assay for RecBCD activity using Functional Data Analysis and Design of Experiments

Adam Winnifrith,[a] Steven R. Brown,[b] Piotr Jedryszek,[c] C. Grant,[b] Philip E. Kay,[d] Adam M. Thomas,[a] Jacob D. Bradbury,[a,e] and Thomas Lanyon-Hogg*[a]

[a]Department of Pharmacology, University of Oxford, OX1 3QT, UK
[b]Synthace, 4th Floor The Westworks, 195 Wood Lane, London, W12 7FQ, UK
[c]Department of Biology, University of Oxford, OX1 3SZ
[d]SAS Institute, Wittington House, Henley Road, Medmenham Marlow, Buckinghamshire, SL7 2EB, UK
[e]Ineos Oxford Institute for Antimicrobial Research, University of Oxford, OX1 3RE, UK
*thomas.lanyon-hogg@pharm.ox.ac.uk

Contents:

Supplementary Figures 3

Supplementary Tables 11

Supplementary Videos 12

Methods 13

References 16

Supplementary Figures

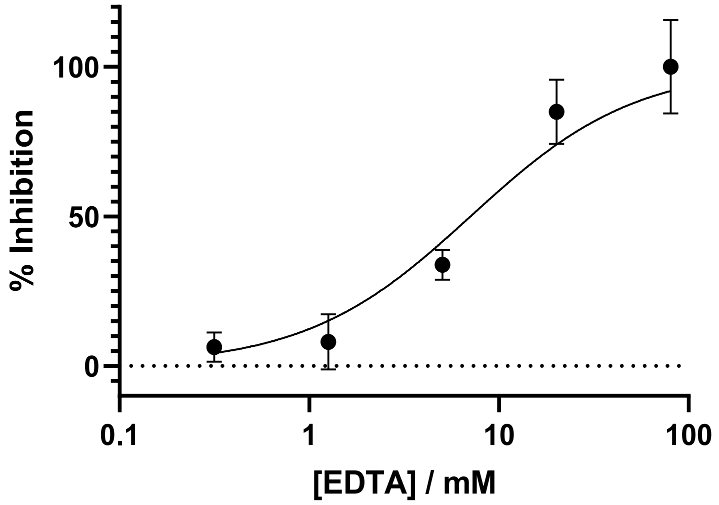

Figure S1. Inhibition of RecBCD by EDTA. A serial dilution of EDTA was added to reaction mixtures containing RecBCD (1.4 nM), ATP (0.7 mM), $MgCl_2$ (14 mM), and potassium ethanoate (36 mM). The reaction was initiated by addition of Lambda DNA (final concentration 2 ng/µL) and stopped after 16 min by addition of EDTA. 20 µL of 1:400 dilution of Quantifluor dye at the concentration provided by the manufacturer was added after 20 min. Inhibition (%) was calculated relative to positive (no RecBCD) and negative (vehicle) controls, and analysed using a sigmoidal four parameter dose-response (variable slope) model. Data represent mean ± standard error of the mean (SEM, n = 3).

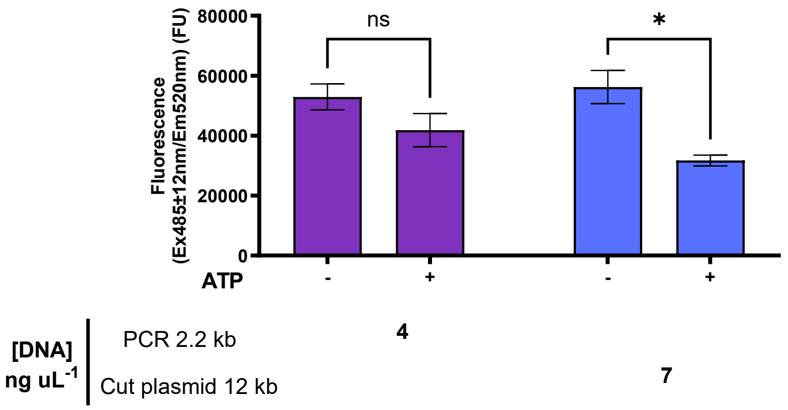

Figure S2. RecBCD activity with alternative DNA substrates. Reactions were prepared with RecBCD (2.4 nM), $MgCl_2$ (10 mM) in reaction buffer as described in methods. The reaction was started by addition of ATP (final concentration 1 mM) and stopped after 11 min by addition of EDTA (final concentration 0.1 M). QuantiFluor (20 µL) was added and fluorescence recorded. DNA substrates were either a PCR product from the pBR322recBC2773D+ plasmid created as described in methods or the pBR322recBC2773D+ plasmid cut with NdeI. Data represents means ± SEM (n = 3), two-way ANOVA main effects (DNA substrate and ATP) and interaction effect, multiple pairwise comparisons using Turkey's t-test with Sidak correction. * and ns represent P <0.05 and nonsignificant, respectively.

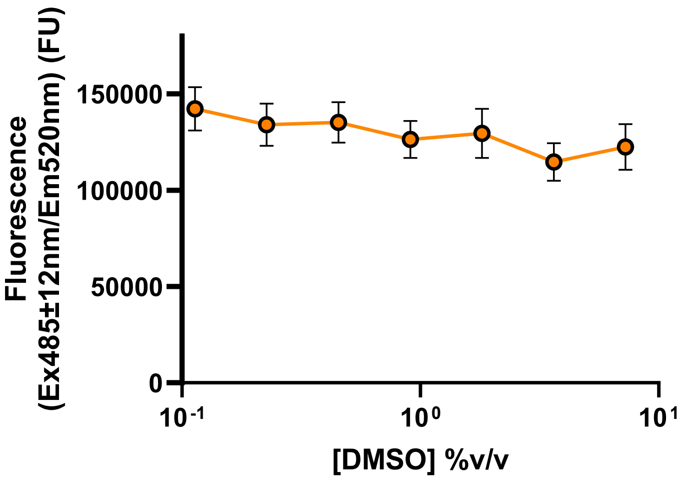

Figure S3. DMSO tolerance of QuantiFluor dsDNA signal. A serial dilution of DMSO was prepared and added to Lambda DNA (final concentration 0.68 mg L^{-1}) and 20 µL of 1:400 dilution of Quantifluor dye at the concentration provided by the manufacturer was added after 20 min. Data represent mean ± SEM (n = 3).

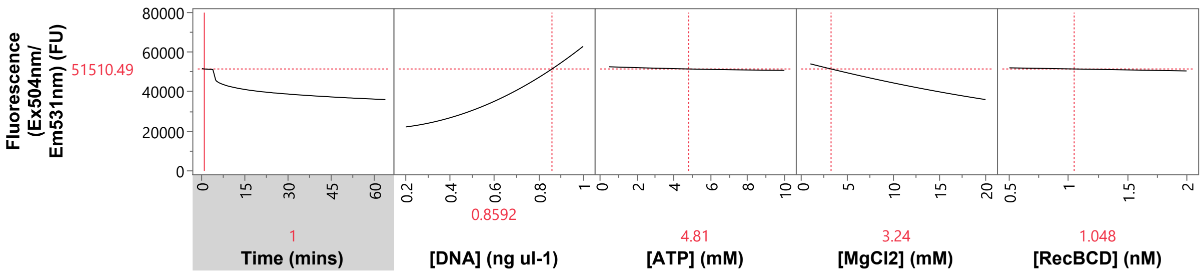

Figure S4. Effect of DNA, ATP, $MgCl_2$, and RecBCD concentrations on the response curve shape of DNA degradation. Reaction conditions were prepared in a 384-well plate using a Dragonfly (SPT) liquid handler. The design criteria covered input factor levels for RecBCD (0.5-2 nM), DNA (0.2-1 ng μL^{-1}), $MgCl_2$ (1-20 mM), and ATP (0.5-10 mM). The reaction was started by addition of ATP (1-10 mM) after 5 min and fluorescence recorded over 60 min. Functional Data Analysis (FDA) was performed on the resulting response curves.

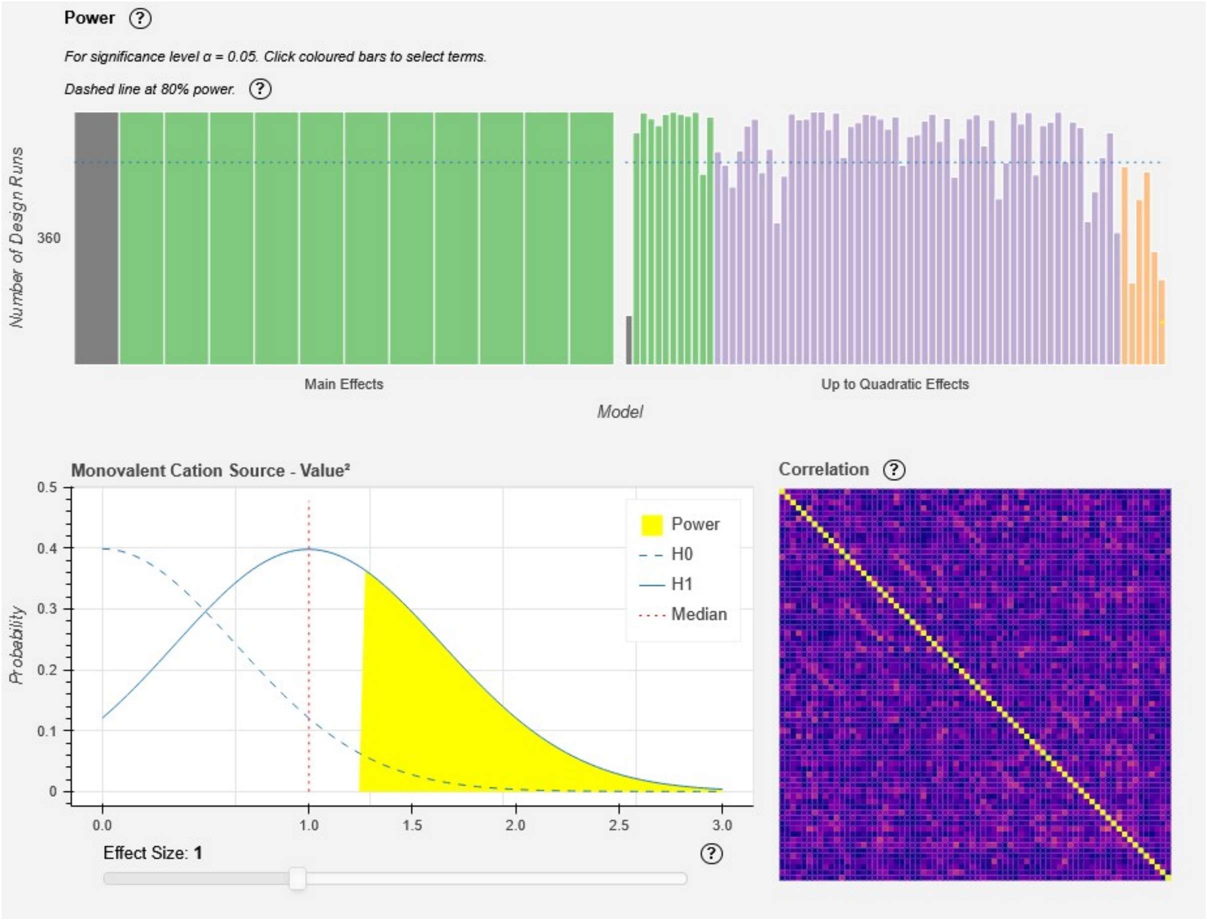

Figure S5. **Statistical power and correlation analysis of high-dimensional 360-run DoE design (Fig. 4).** (Top) Bar chart illustrating the power to detect main effects (green), quadratic effects (purple), and higher-order interactions (orange) at a 5% significance level. The dashed line indicates 80% power. (Bottom left) Plot of statistical power under the null (H0) and alternative (H1) hypotheses, assuming normality, by showing the degree of overlap between the H0 and H1 distributions at varying effect sizes. (Bottom right) Correlation matrix visualising pairwise correlations among the design factors, aiding in the identification of any strong inter-factor dependencies that may impact the model.

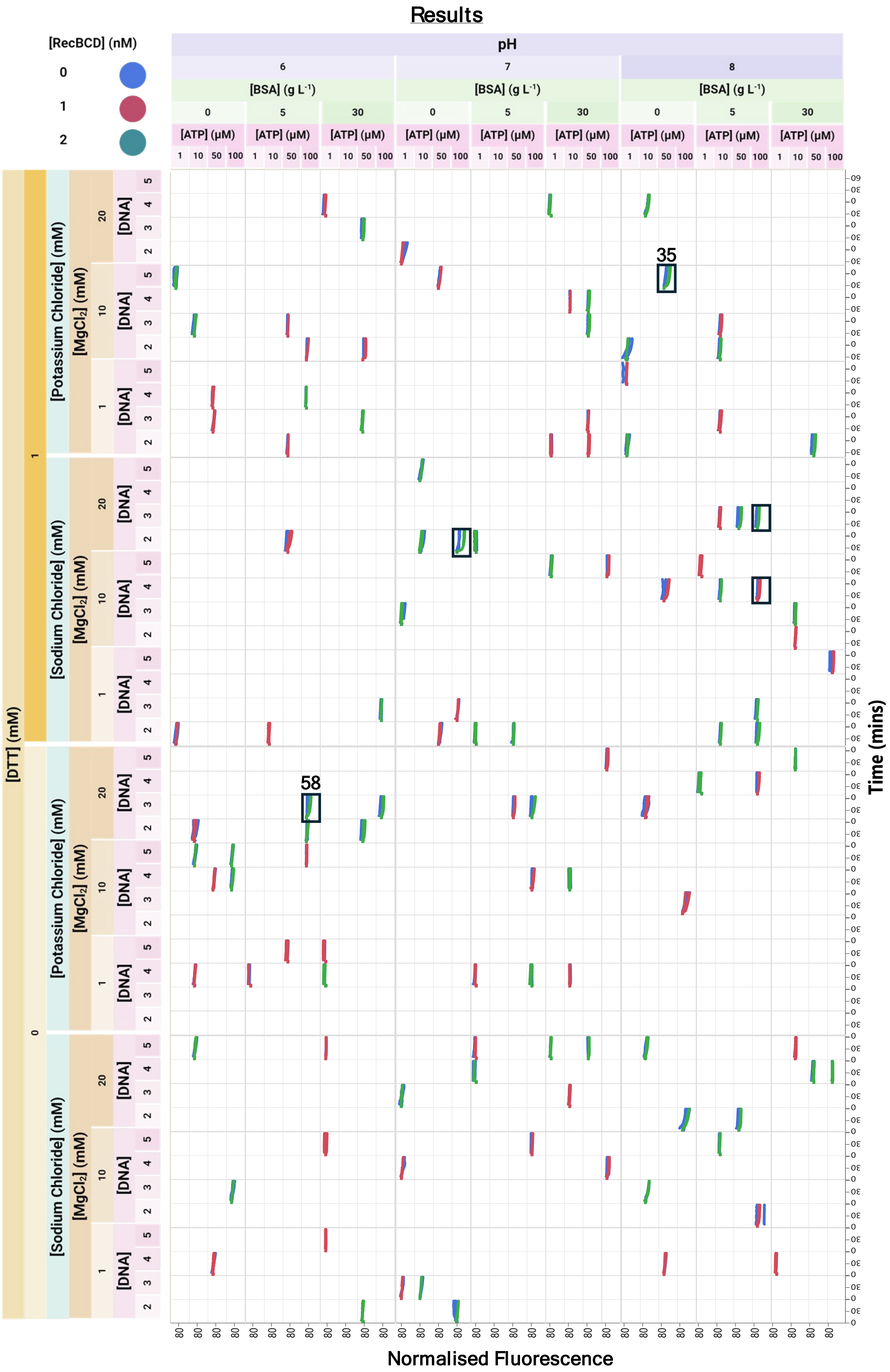

Figure S6. Fluorescence response curves from high-dimensional experimentation exploration of RecBCD assay (Fig. 4). Normalised fluorescence against time is displayed for each tested condition, grouped by the variables that define the condition. Conditions with highest Z' are boxed in bold.

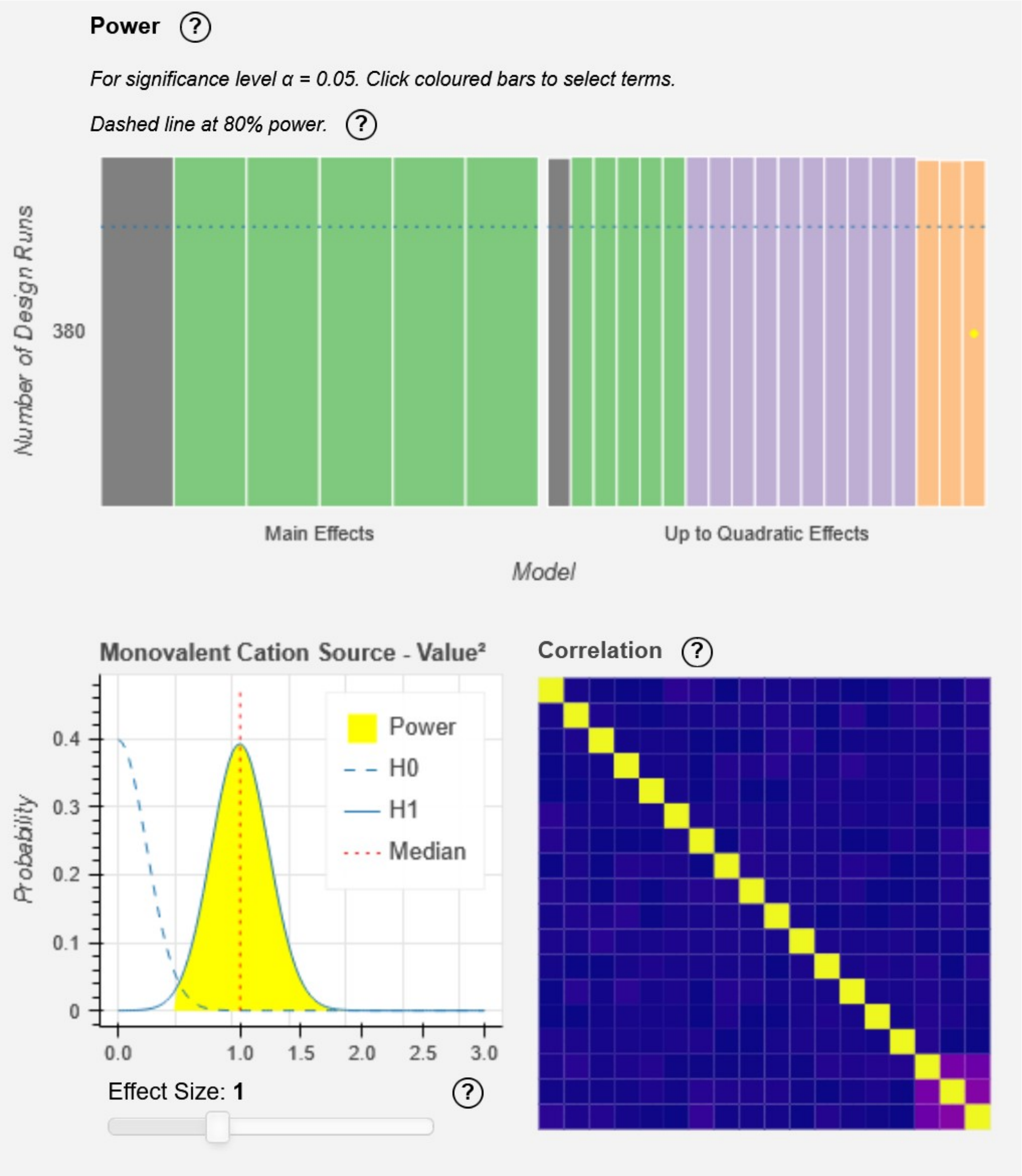

Figure S7. Statistical power and correlation analysis of localised 384-run DoE design (Fig. 5). (Top) Bar chart illustrating the power to detect main effects (green), quadratic effects (purple), and higher-order interactions (orange) at a 5% significance level. The dashed line indicates 80% power. (Bottom left) Plot of statistical power under the null (H0) and alternative (H1) hypotheses, assuming normality, by showing the degree of overlap between the H0 and H1 distributions at varying effect sizes. (Bottom right) Correlation matrix visualising pairwise correlations among the design factors, aiding in the identification of any strong inter-factor dependencies that may impact the model.

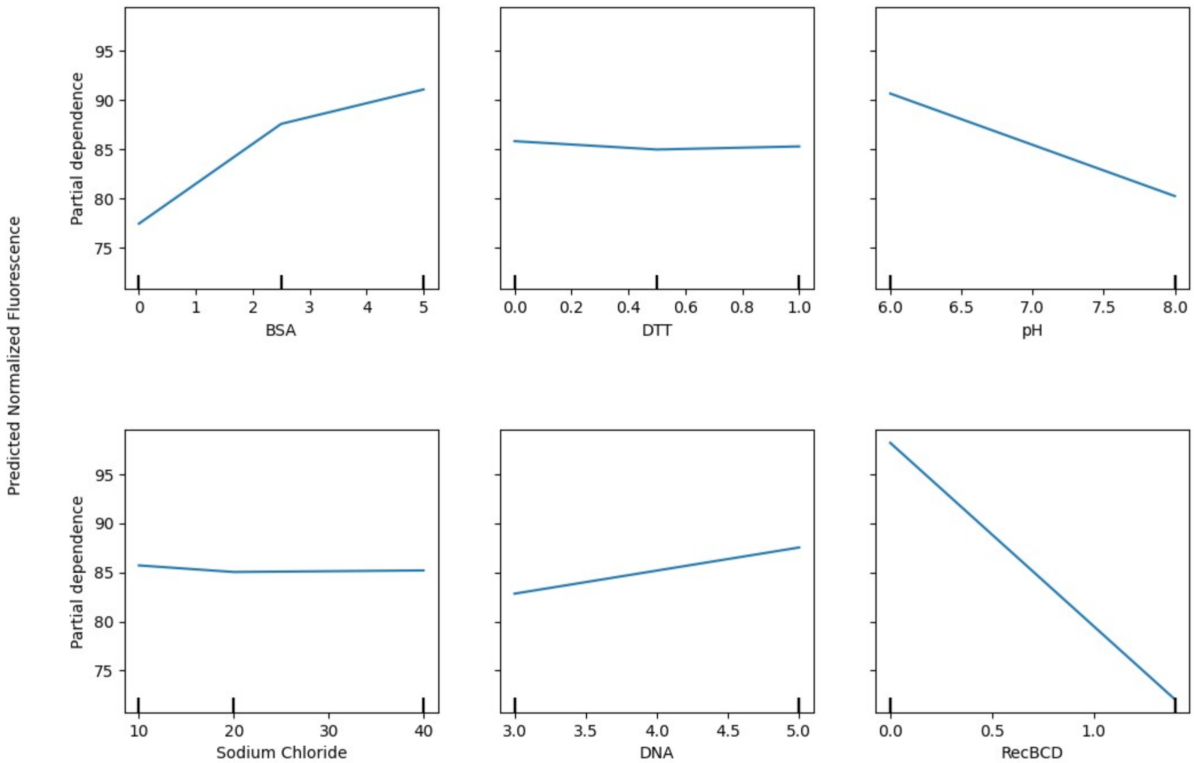

Figure S8: Partial dependency plots from DoE analysis of 'hit' assay conditions. A random forest model was trained to predict the normalised fluorescence at time = 30 min, indicating RecBCD, BSA, and pH had feature importances of 53%, 23%, and 16%, respectively (R^2 = 0.92, root mean squared error {RMSE} = 5.5)

Supplementary Tables

Table S1. Design of experiment to assess factors affecting RecBCD reaction progression (Fig. 3).

Run Order	[DNA] (ng µL^{-1})	[ATP] (mM)	[$MgCl_2$] (mM)	[RecBCD] (nM)	Volume (µL)
1	1	5.25	10.5	0.5	100
2	1	5.25	10.5	2	100
3	0.2	5.25	1	0.5	100
4	0.2	0.5	10.5	0.5	100
5	0.2	10	1	2	100
6	1	5.25	10.5	2	100
7	1	5.25	10.5	0.5	100
8	0.2	5.25	10.5	2	100
9	0.2	0.5	20	2	100
10	0.2	5.25	1	0.5	100
11	1	10	1	2	100
12	0.2	10	10.5	0.5	100
13	0.2	5.25	10.5	2	100
14	0.2	0.5	1	2	100
15	1	5.25	10.5	2	100
16	0.2	5.25	20	0.5	100
17	1	0.5	20	0.5	100
18	1	10	20	2	100
19	0.2	10	10.5	0.5	100
20	0.2	0.5	10.5	0.5	100
21	0.2	10	20	2	100
22	0.2	5.25	10.5	2	100
23	1	0.5	20	2	100
24	1	0.5	1	2	100
25	0.2	5.25	20	0.5	100
26	1	0.5	1	0.5	100
27	1	10	1	0.5	100
28	1	10	20	0.5	100

Table S2. Design of high-dimensional experiment to explore RecBCD assay signal window (Fig. 4, additional Excel file).

Table S3. Design of experiment to explore 'hit' conditions for RecBCD activity (Fig. 5, additional Excel file).

Supplementary Videos

Each video shows the interactive JMP Prediction Profiler output from functional data analysis (FDA) of a DoE experiment. The output of each model is the predicted fluorescence response against time curve (left-hand graph). The input factors for the model are the right-hand graphs, with the vertical red lines indicating the selected levels of each input factor (the selected numerical value for each level is shown in red below the graph). The levels of each input factor are varied by moving the corresponding vertical red lines, and the predicted fluorescence response against time curve changes depending on the selected combination.

The graph above each individual input factor shows how the predicted fluorescence response *at the currently selected timepoint on the left-hand (predicted fluorescence against time) graph* changes in response to that input factor, at the currently selected levels of the other input factors. FDA can therefore not only show how the response curve changes as a result of different input factor combinations, but also how changing one input factor affects the effect of other factors on the response curve.

Video S1. Exploration of the FDA Prediction Profiler output from the experiment shown in Figure 3. Input factors DNA, ATP, $MgCl_2$, and RecBCD.

Video S2. Exploration of the FDA Prediction Profiler output from the experiment shown in Figure 5. Input factors BSA, DTT, DNA, pH, sodium chloride, and RecBCD.

Methods

Abbreviations

BSA (bovine serum albumin); DMSO (dimethyl sulfoxide); DTT (dithiothreitol); EDTA (ethylenediaminetetraacetic acid); SSB (single-strand DNA-binding protein).

Biochemical Reagents

Chemical reagents, solvents and buffer solutions were purchased from Sigma Aldrich (Missouri, USA) or ThermoFisher (Massachusetts, USA). BSA was purchased from ThermoFisher. QuantiFluor ONE (E4891), QuantiFluor ONE dsDNA System (E4871) and QuantiFluor® dsDNA System (E2671) were purchased from Promega (Wisconsin, USA). RecBCD M0345L and Lambda DNA were purchased from New England Biolabs (NEB; Massachusetts, USA).

Bacterial Strains and Growth

The bacterial strain used for plasmid purification was *E. coli* ΔrecBCD2731<kan> argA21 hisG4 met recF143 rpsL31 galK2 xyl-5 lambda- F- (pBR322recBC2773D+; ampR).[1,2] A single colony from an LB agar plate was grown overnight in high salt LB supplemented with ampicillin (100 µg mL^{-1}) at 37 °C, 200 rpm, with a 1:10 ratio of liquid to air in the culture container.

Plasmid Purification

Plasmid purifications were performed using NEB miniprep kits, Qiagen Maxi Prep kits, and Zymo Maxi Prep kits as per the manufacturer's protocol.

PCR

A standard PCR reaction of the pBR322recBC2773D+ plasmid was performed as per the manufacturer's protocol (NEB). Primers were designed using Benchling (forward: ggggctgaacgcgctggatatc and reverse: tgccagcaacttcgctacggtg). Reactions were performed with 5 µM primers, 1 ng of plasmid, 25 µL of Q5Master Mix (NEB), and 19 µL of water. Cycle settings of 10 s at 98 °C, 30 s at 72 °C, 60 s at 72 °C were used.

Manual RecBCD Assay (Fig. 2)

The reaction buffer was either NEB Buffer 4 (50 mM potassium ethanoate, 20 mM tris ethanoate, 10 mM magnesium ethanoate, 1 mM DTT, pH 7.9) or a prepared 'Reaction Buffer' of (50 mM potassium ethanoate, 20 mM Tris, pH 7.9) and a separate addition of $MgCl_2$, depending on whether the experiment required variation of Mg^{2+} concentration.

Reagent Preparation: All reagents were thawed on ice and centrifuged for 30 s using a microcentrifuge. Reaction conditions were prepared in microcentrifuge tubes (Eppendorf) and transferred to a black 384-well plate (Corning). **Reaction Initiation:** The reaction was started by the addition of ATP or DNA substrate at the specified concentrations (see figure legends for details). **Endpoint Measurements:** For endpoint measurements, EDTA was added at the specified time to a final concentration of 0.1 M. QuantiFluor dsDNA dye was then added at 1:400 dilution unless otherwise stated and at the specified volume. **Kinetic Measurements:** For kinetic reads, QuantiFluor

was added at 1:400 dilution before the start reagent. **Fluorescence Measurement:** Fluorescence was measured using a Clariostar plate reader (BMG Labtech) at either Ex 485 nm/Em 520 nm or Ex 504 nm/Em 531 nm.

Automated RecBCD Assay (Fig. 3-5)

Reagent Preparation: All buffer components and reaction components were prepared, diluted, and placed onto the Dragonfly (SPT) deck. Components that were not volatile or sensitive were dispensed first. **Automated Liquid Handling:** The Dragonfly automated liquid handler prepared all assay samples in a 384-well plate. **Pre-Reaction Measurements:** After dispensing, the plate was centrifuged and read using the FLUROstar plate reader to quantify fluorescence, with readings taken every minute for 5 min. **Reaction Initiation:** The plate was then transferred back to the Dragonfly, and ATP was dispensed into all wells. **Kinetic Measurements:** The plate was transferred back to the FLUROstar for fluorescence measurement every minute for 55 min.

Design of Experiments and Functional Data Analysis for RecBCD Assay

Experimental Designs

JMP (SAS Institute Inc., Cary, NC, USA) and Synthace (White City, London, UK) software packages were used to generate constrained D-Optimal and space-filling designs. The range of levels tested for each factor was determined based on prior literature, and the number of repeats chosen by balancing the number of 384-well plates there was capacity to analyse and the desired statistical power.

Model Building and Statistical Analysis

All models were built in JMP. Single response analysis used standard least squares models initially including all main, quadratic, and interaction effects for each factor involved. Variable selection was then performed using backwards selection whereby model parameters with the largest p-values were sequentially removed with a stopping rule of a p-value of 0.10. [3]

The significance of each model effect was assessed by conducting a Partial F Test to test the null hypothesis that the parameters associated with the effect were zero in the context of a model that also included other effects. A significance threshold of p=0.05 was used.

Z' Calculation

Z' was calculated using the following equation:

$$Z' = 1 - \frac{3(\sigma_p + \sigma_n)}{|\mu_p - \mu_n|}$$

Where:

σ_p = Standard deviation of the positive control

σ_n = Standard deviation of the negative control

μ_p = Mean of the positive control

μ_n = Mean of the negative control

Z' was calculated for each experimental condition and timepoint, with the mean Z' between 25-40 min used for ranking conditions.

Functional Data Analysis

Kinetic data were analysed using Functional Data Analysis (FDA) in JMP.[4] A smoothed model of the fluorescence-time curve for each experimental run was constructed using a wavelet model for each condition. A mean fluorescence-time curve across all experimental runs was calculated, and each individual model was decomposed into this mean curve and various linear combinations of Principal Component shape curves using Functional Principal Component Analysis (FPCA). A generalized regression model was used to understand how input factors and DoE factors influenced the Functional Principal Components (FPCs) of the wavelet models. This regression model utilized the Akaike information criterion corrected for small samples as the stopping criterion.

Statistical Analysis

A Random Forest algorithm was employed to predict fluorescence measurements at t = 30 min using a dataset comprising of input factors pH, BSA, DNA, RecBCD, DTT, and sodium chloride. The dataset was split into 80% training and 20% test sets to evaluate model performance. Feature importance analysis was conducted to identify the relative contribution of each feature to the model's predictions. Model performance was assessed using metrics R^2 and root mean squared error (RMSE).

References

1 S. K. Vester, R. Rahikainen, I. N. A. Khairil Anuar, R. A. Hills, T. K. Tan and M. Howarth, SpySwitch enables pH- or heat-responsive capture and release for plug-and-display nanoassembly, *Nat Commun*, 2022, **13**, 3714.
2 S. K. Amundsen, A. F. Taylor, M. Reddy and G. R. Smith, Intersubunit signaling in RecBCD enzyme, a complex protein machine regulated by Chi hot spots, *Genes Dev.*, 2007, **21**, 3296–3307.
3 J. Sall, A. Lehman, M. Stephens and S. Loring, *JMP Start Statistics: A Guide to Statistics and Data Analysis Using JMP*, SAS Institute Inc, Cary, North Carolina, Sixth edition., 2017.
4 J. O. Ramsay and B. W. Silverman, *Functional Data Analysis*, Springer, New York, 2nd ed., 2005.
